# Comparison of photodamage in non-pigmented and pigmented human skin equivalents exposed to repeated ultraviolet radiation to investigate the role of melanocytes in skin photoprotection

**DOI:** 10.3389/fmed.2024.1355799

**Published:** 2024-04-18

**Authors:** Paola De Los Santos Gomez, Lydia Costello, Kirsty Goncalves, Stefan Przyborski

**Affiliations:** ^1^Department of Biosciences, Durham University, Durham, United Kingdom; ^2^Reprocell Europe Ltd., Glasgow, United Kingdom

**Keywords:** UV radiation, human skin equivalents, pigmentation, photodamage, photoprotection, melanocytes

## Abstract

**Introduction:**

Daily solar ultraviolet (UV) radiation has an important impact on skin health. Understanding the initial events of the UV-induced response is critical to prevent deleterious conditions. However, studies in human volunteers have ethical, technical, and economic implications that make skin equivalents a valuable platform to investigate mechanisms related to UV exposure to the skin. *In vitro* human skin equivalents can recreate the structure and function of *in vivo* human skin and represent a valuable tool for academic and industrial applications. Previous studies have utilised non-pigmented full-thickness or pigmented epidermal skin equivalents to investigate skin responses to UV exposure. However, these do not recapitulate the dermal-epidermal crosstalk and the melanocyte role in photoprotection that occurs *in vivo*. In addition, the UV radiation used in these studies is generally not physiologically representative of real-world UV exposure.

**Methods:**

Well-characterised pigmented and non-pigmented skin equivalents that contain human dermal fibroblasts, endogenous secreted extracellular matrix proteins (ECM) and a well-differentiated and stratified epidermis have been developed. These constructs were exposed to UV radiation for ×5 consecutive days with a physiologically relevant UV dose and subsequently analysed using appropriate end-points to ascertain photodamage to the skin.

**Results:**

We have described that repeated irradiation of full-thickness human skin equivalents in a controlled laboratory environment can recreate UV-associated responses *in vitro,* mirroring those found in photoexposed native human skin: morphological damage, tanning, alterations in epidermal apoptosis, DNA lesions, proliferation, inflammatory response, and ECM-remodelling.

**Discussion:**

We have found a differential response when using the same UV doses in non-pigmented and pigmented full-thickness skin equivalents, emphasising the role of melanocytes in photoprotection.

## 1 Introduction

The skin is a complex organ that covers the external surface of the human body and acts as an interface between the internal environment and external environmental stressors contributing to maintaining homeostasis. The skin is a physical barrier that protects against water and electrolyte loss, UV radiation and other physical, chemical, and biological exogenous stressors (1). Solar radiation, air pollution, tobacco smoke, temperature and humidity are environmental stressors known to have a primary role in skin health (2). Solar UV radiation on human skin has been extensively studied, and besides its beneficial effect on vitamin D synthesis, different deleterious effects have been characterised in irradiated human skin: sunburn (also erythema), pigmentation, inflammatory responses, photoageing, and DNA damage leading to mutations and skin cancer (3–25).

Due to the complexity of biological effects induced by various exposure conditions (spectrum, wavelength, doses, skin penetration), technical (skin biopsy collection) and ethical (UV doses and courses) issues are often present when studying the early events in UV exposure to skin in human volunteers. Studies in animal models also involve ethical constraints; moreover, they do not accurately replicate the biological response of native human skin (26). In contrast, quantitative and qualitative approaches to study UV-induced effects on skin and relevant biomarkers can be used *in vitro* with the development of skin equivalents (27). However, the non-physiological relevant UV dose, lack of dermal compartment (28–33) or non-pigmented systems (34–39) are strategies that do not accurately replicate the UV-induced response of human skin.

Epidermal melanin pigment is recognised for its role in photoprotection against UV radiation. The crosstalk between fibroblasts, keratinocytes and melanocytes is required for melanogenesis, melanin transfer, and the correct melanin organisation as supranuclear caps that protect nuclear DNA from UV induced damage (40–49). Such processes are poorly recapitulated in many previous *in vitro* approaches that are more limited representations of native skin. Therefore, different biological responses compared to those identified in irradiated native human skin can be expected due to structural differences of the skin equivalent. Moreover, the inclusion of exogenous collagen-based hydrogels, a widely used strategy for the generation of the dermal compartment in the pigmented full-thickness skin equivalents and UV-induced skin equivalents (38, 50–54) could also limit the recapitulation of the native human ECM structure (55). This is likely because differences in ECM composition and the addition of exogenous components could influence pigmentation and UV responses when investigating UV exposure to human skin, photoageing and pigmentation *in vitro*.

To address these limitations, we have developed a platform for evaluating the effects of physiologically relevant quantities of UV irradiation delivered in a controlled manner to the surface of a robust human full-thickness skin equivalent (56). In this study, we monitored relevant end-points in UV-irradiated human skin and recorded the influence of melanocytes and melanin photoprotection by analysis of morphological damage, proliferation, apoptosis, DNA lesions, pigmentation-related changes in the epidermis, and inflammatory and ECM remodelling demonstrating the ability to more accurately recapitulate features of photoexposed native human skin in the laboratory.

## 2 Materials and methods

### 2.1 Cell culture

Commercially available cells used to create human skin equivalents include human neonatal dermal fibroblasts #1366356 and #1366434 (HDFn, Thermo Fisher Scientific), human neonatal epidermal keratinocytes #1803415, #1817888, #2018512, and #2286109 (HEKn, Thermo Fisher Scientific, Loughborough, United Kingdom), and darkly pigmented human neonatal epidermal melanocytes #2077650 (HEMn-DP, Thermo Fisher Scientific). HDFn were maintained in Dulbecco’s Modified Eagle Medium (DMEM, Thermo Fisher Scientific) supplemented with fetal bovine serum (FBS, Thermo Fisher Scientific) and L-glutamine 200 mM (Thermo Fisher Scientific); HEKn were maintained in EpiLife medium, (Thermo Fisher Scientific), supplemented with human keratinocyte growth supplement (HKGS, Thermo Fisher Scientific); and HEMn were maintained in Medium 254^®^ (Thermo Fisher Scientific), supplemented with human melanocyte growth supplement (HMGS, Thermo Fisher Scientific), at 37°C in a 5% CO_2_ in a humidified environment following the supplier’s instructions.

### 2.2 Skin equivalent generation

The generation of human-pigmented skin equivalents was modified from a previously described methodology (56, 57). HEMn were trypsinised and seeded simultaneously with HEKn onto a 21-day matured dermal compartment at a ratio of 1:10 (melanocytes:keratinocytes). Cultures were maintained in submerged culture for 48 h and raised to the air-liquid interface (ALI) to promote keratinocyte differentiation and stratification for a further 14 days prior to use in experiments. Three human skin equivalents per condition and three independent technical replicates were developed.

### 2.3 UV exposure

A Bio-Sun UV irradiator (LTF Labortechnik GmbH & Co. KG, Wasserburg am Bodensee, Germany) was used to expose the human skin equivalents to UV radiation. The calculation of a physiologically relevant UV dose has been previously described (56). Briefly, skin equivalents were transferred to a new sterile 6-well plate with 4 mL Dulbecco’s phosphate-buffered saline (DPBS) each to keep them in ALI. They were rinsed once in DPBS before irradiation to remove culture media components such as phenol red to avoid the formation of phototoxic products and interference with UV-induced pigmentation. Non-irradiated skin equivalents were rinsed in DPBS and left in the cell culture hood for the duration of the exposure. Human skin equivalents were irradiated once a day for 5 consecutive days. Exposure to a solar-simulated UV dose of 3.3 J cm^−2^ (96.5% UVA, 3.5% UVB) lasted approximately 15 min. Following irradiation, the skin equivalents were transferred to their culture media and incubated until the subsequent irradiation 24 h later or harvested 48 h after the last irradiation.

### 2.4 Human skin samples

Full-thickness 4 mm skin biopsies were obtained from the photoexposed dorsal forearm and photoprotected buttock of young, healthy, female volunteers. Skin biopsies were collected by Procter and Gamble (Cincinnati, OH, United States) under an IRB-approved clinical protocol in compliance with local laws and regulations. Participants signed informed consent and were compensated for their participation. Skin samples were transferred to Durham University where they were processed as previously described (58), embedded in paraffin wax, and histologically stained in the same manner as human skin equivalents.

### 2.5 Individual typological angle and melanin index readings

Individual typological angle (ITA) and melanin index (MI) measurements were obtained using a colourimeter, the SkinColorCatch (Delfin Technologies, Surrey, United Kingdom). The SkinColorCatch is a clinical instrument and colourimeter that is extensively used to determine facets of skin tone reliably in both *in vitro* (56) and *in vivo* settings (59–62). The readings were repeated three times in different surface areas to get an average value of the parameter.

### 2.6 Conditioned media preparation for analysis

Secretion of inflammatory and ECM remodelling proteins was analysed in conditioned medium of skin equivalents before UV exposure, during UV exposure (before the fourth dose), and after UV exposure (48 h post-UV), corresponding to days 15, 18 and 21 at ALI. Four hundred microlitres of media was collected from and frozen at −80°C. Analysis of the medium was performed by Eve Technologies (Calgary, Canada) using the Human Cytokine Proinflammatory Focused 15-Plex, and the Human MMP and the TIMP Discovery Assay^®^ Array for Cell Culture and non-blood samples. Three technical repeats per time point per condition of two independent experiments were analysed.

### 2.7 Paraffin wax embedding

Skin equivalents and human skin samples were fixed in 4% paraformaldehyde and gradually dehydrated in ethanol and incubated in Histoclear (Scientific Laboratory Supplies, Nottingham, United Kingdom), Histoclear:paraffin wax and 100% paraffin wax (CellPath, Newton, United Kingdom). Samples were embedded in plastic moulds (Solmedia, Shrewsbury, United Kingdom) with paraffin wax. Paraffin wax blocks were sectioned at 5 μm using a rotary manual microtome Leica RM2125RT (Leica Biosystems, Nussloch, Germany) with MB DynaSharp microtome blades (Thermo Fisher Scientific). Transverse sections were mounted onto charged Superfrost Plus microscope slides (Thermo Fisher Scientific).

### 2.8 Histological staining and imaging

Skin equivalents and human skin samples were deparaffinised in Histoclear (Scientific Laboratory Supplies) and gradually rehydrated in ethanol.

For hematoxylin & eosin (H&E) staining, samples were incubated in Mayer’s hematoxylin (Sigma-Aldrich) for 5 min followed by alkaline ethanol for 30 s. Slides were dehydrated in ethanol before incubation with eosin (Sigma-Aldrich) for 30 s and further dehydrated. Finally, slides were cleared in Histoclear and mounted with Omnimount (Scientific Laboratory Supplies).

Fontana Masson melanin staining was achieved using a commercially available kit (Abcam, Cambridge, United Kingdom, ab150669) following the manufacturer’s instructions.

Histological images were captured using a Leica ICC50 high-definition camera (Leica Microsystems, Wetzlar, Germany) mounted onto a DM500 Leica microscope (Leica Microsystems). Images were processed using the Fiji software (63).

### 2.9 Immunofluorescence staining and imaging

Sections were deparaffinised in Histoclear and gradually rehydrated in ethanol. Antigen retrieval was performed in citrate buffer pH 6 (Sigma-Aldrich) at 95°C for 20 min, followed by blocking and permeabilisation for 1 h with 20% neonatal calf serum (NCS, Sigma-Aldrich) in 0.4% Triton X-100 (Sigma-Aldrich) in phosphate-buffered saline (PBS). Samples were incubated overnight at 4°C in primary antibody diluted in blocking buffer (Ki-67, Abcam, ab16667, 1:100; Cyclobutane Pyrimidine Dimers, Cosmo Bio United States, Carlsbad, California, United States, CAC-NM-DND-001, 1:1000). Slides were washed three times in PBS and incubated with the secondary antibody diluted in blocking buffer for 1 h at room temperature (donkey anti-rabbit Alexa Fluor 594, donkey anti-mouse Alexa Fluor 488, Thermo Fisher Scientific, 1:1000; Hoechst 33342 Fluorescent Stain, Thermo Fisher Scientific, 1:10000) and washed three times in PBS. Slides were mounted using Vectashield antifade mounting medium (Vector Laboratories, Peterborough, United Kingdom).

The fluorescent images were captured using Zeiss 880 with Airyscan confocal microscope (Carl Zeiss AG, Oberkochen, Germany) using Zeiss Zen software. Images were processed using the Fiji software.

### 2.10 Epidermal whole-mount staining and imaging

To visualise the distribution of melanocytes across the epidermis, the epidermal layer was separated from the underlying dermis through chemical digestion. Five millimetre punch biopsies were taken from the skin equivalents using a 5 mm Kai sterile dermal biopsy punch (Selles Medical Ltd., East Yorkshire, United Kingdom). Punch biopsies were placed *stratum corneum* down in a solution of 3.8% ammonium thiocyanate (Sigma-Aldrich) in PBS at room temperature for 20 min.

The epidermis was peeled from the dermis using forceps and washed twice in PBS. The epidermal sample was fixed in a 1:1 solution of methanol (Thermo Fisher Scientific) and acetone (Thermo Fisher Scientific) for 20 min at −20°C. Samples were blocked and permeabilised for 1 h in 20% NCS and 0.4% Triton X-100 in PBS. Samples were then incubated at room temperature for 2 h in primary antibody diluted in blocking buffer (TYRP1, Abcam, ab190709, 1:100). Samples were washed three times in PBS for 10 min each, incubated with secondary antibody diluted in blocking buffer for 1 h at room temperature (donkey anti-mouse Alexa Fluor 488, 1:1000), and washed three times in PBS.

Epidermal sheets were mounted using Vectashield antifade mounting medium with the *stratum basale* facing up to allow imaging of a large area of the *stratum basale* and provide reliable information as to melanocyte density. The fluorescent images of TYRP-1 positive melanocytes located in the *stratum basale* were captured using Zeiss 880 with Airyscan confocal microscope using Zeiss Zen software. Images were processed using the Fiji software.

### 2.11 TUNEL assay and imaging

The detection of apoptotic cells in the skin equivalents was achieved through the TdT-mediated dUTP-biotin nick end labelling (TUNEL) assay. The DeadEnd^™^ Fluorometric TUNEL System (Promega United Kingdom Ltd., Hampshire, United Kingdom) was used to detect cell nuclei in apoptotic cells in wax-embedded skin sections following the manufacturer’s instructions. The fluorescent images were captured using Zeiss 880 with Airyscan confocal microscope using Zeiss Zen software. Images were processed using the Fiji software.

### 2.12 Biometric analysis

#### 2.12.1 Melanocyte density

Melanocyte density was quantified using images of TYRP1-stained epidermises. One punch biopsy per skin equivalent was stained, and three images were taken in different areas of the punch biopsy using confocal fluorescence microscopy. The multipoint tool in the Fiji software was used to count the number of TYRP1-positive cells. The melanocyte density was calculated using the number of melanocytes divided by the area of the image (average surface of 0.501958 mm^2^ per sample).

#### 2.12.2 Epidermal proliferation

Epidermal proliferation was quantified using images of Ki67-stained skin equivalent sections. The multipoint tool in the Fiji software was used to count the number of Ki67-positive nuclei and the total number of Hoechst-stained nuclei in the basal layer, which were used to calculate the percentage of Ki67-positive cells in the epidermis. One section per skin equivalent was stained, and three random images were taken per section at 20x magnification.

#### 2.12.3 Epidermal apoptosis

Cell death in the epidermis was quantified using images of TUNEL-immunostained skin equivalents. The multipoint tool in the Fiji software was used to count the number of TUNEL-positive nuclei and the total number of Hoechst-stained nuclei, which were used to calculate the percentage of TUNEL-positive cells in the epidermis. One section per skin equivalent was stained, and three random images were taken per section at 40x magnification.

#### 2.12.4 Epidermal DNA damage

UV-induced DNA damage was quantified using images of cyclobutane pyrimidine dimers (CPD)-immunostained skin equivalents. One section per skin equivalent was stained, and three random images were taken per section at 20x magnification. All the sections were stained together. Acquisition parameters were kept constant within the imaging to ensure comparable signal levels. Image analysis was performed using Fiji. In each image, the epidermis was selected for analysis. Hoechst-stained epidermal nuclei were identified in each microscopic field by the IsoData thresholding method and image segmentation. Nuclear CPD-mean fluorescence intensities were calculated as percentage of maximal intensity (65,535 grey values).

### 2.13 Statistics

Student *t*-test and two-way ANOVA analyses were conducted using GraphPad Prism version 9 software for Windows (GraphPad Software, La Jolla, California, United States). Statistical differences were noted as *, **, *** or **** corresponding to *p* < 0.05, <0.01, <0.001, <0.0001, respectively. No notes above bars describe no statistical difference.

## 3 Results

### 3.1 Melanin protection against UV-induced histological damage in the epidermis of repeatedly irradiated skin equivalents

**Figure 1:**
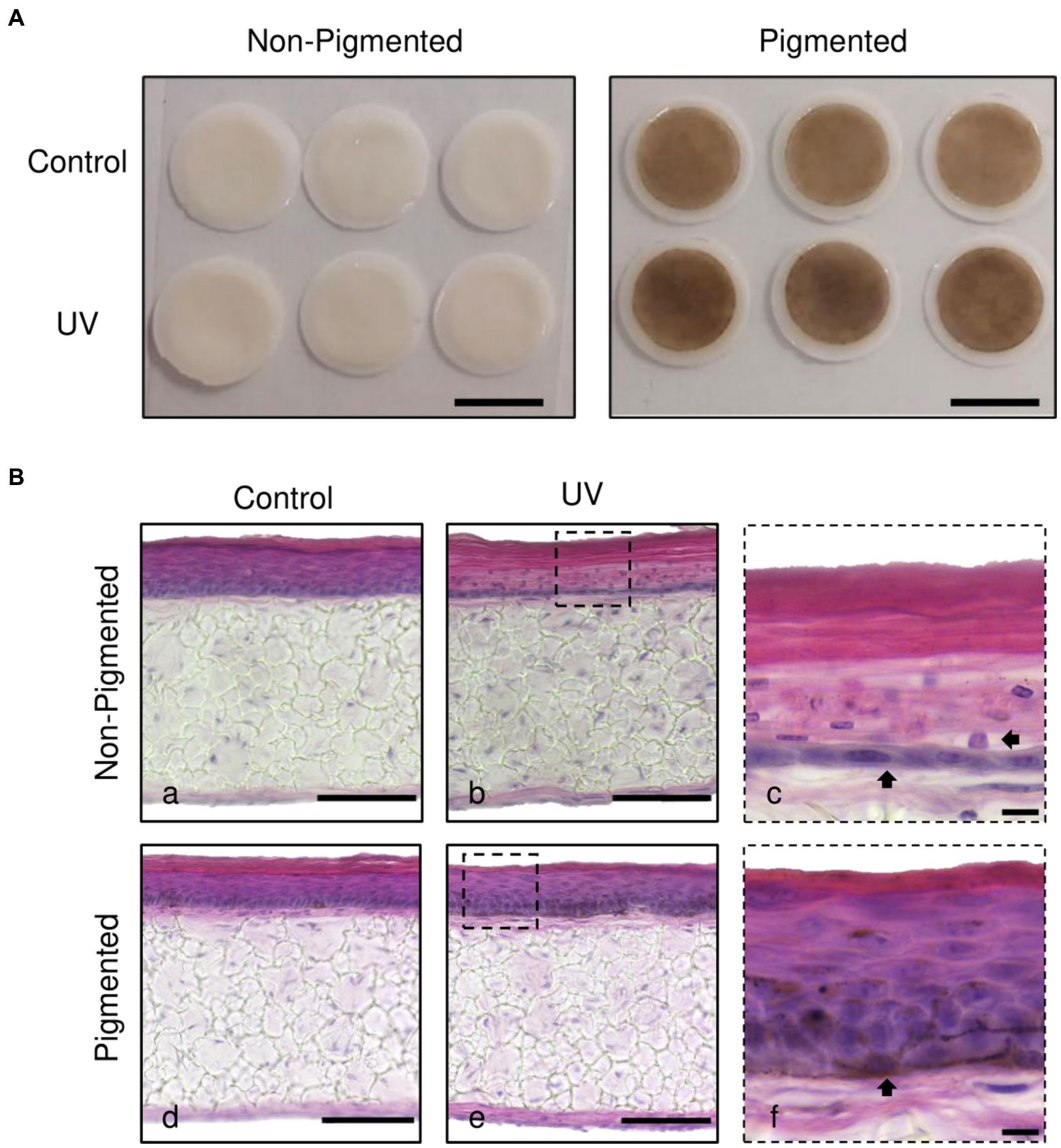
Melanocyte protection against UV-induced structural epidermal damage after repeated UV exposure. Gross appearance **(A)** and histological analysis **(B)** of cross-sections of untreated **(Ba,d)** and repeatedly irradiated skin equivalents **(Bb,e)**. UV-induced skin equivalents were irradiated with 3.3 J cm^−2^ (96.5% UVA and 3.5% UVB) for 5 consecutive days from day 15 to 19 at ALI and harvested 48 h later. Higher magnification images of repeated UV exposure to skin equivalents show morphological alterations in the epidermis of irradiated non-pigmented skin equivalents (**Bc**, arrows). In contrast, irradiated pigmented skin equivalents **(Bf)** demonstrated normal differentiation and stratification of the epidermis despite photoexposure. A melanocyte is prominently visible in the basal layer (**Bf**, arrow). Micrographs represent ×3 independent experiments with ×3 skin equivalents per condition. Scale bars: 1 cm **(A)**, 50 μm **(Ba,b,d,e)**, 10 μm **(Bc,f)**.

Histological analysis was performed to investigate the photoprotective role of melanin in irradiated skin equivalents. Macroscopically, skin equivalents show visible differences in colour between non-pigmented and pigmented skin equivalents. A homogenous pigmentation is observed in pigmented skin equivalents, with a visible tanning effect in the irradiated skin equivalents (Figure 1A). Histological analysis of the untreated skin equivalents displayed correct epidermal differentiation and stratification in non-pigmented and pigmented skin equivalents (Figure 1Ba,d). In contrast, irradiated non-pigmented skin equivalents (Figure 1Bb,c) showed structural epidermal UV-induced damage 48 h post-UV. Repeatedly irradiated non-pigmented skin equivalents exhibited thinning of the viable epidermis, *stratum corneum* thickening and parakeratosis (Figure 1Bb,c). In contrast, repeatedly irradiated pigmented skin equivalents demonstrated normal epidermal differentiation and stratification (Figure 1Be,f), showing unaffected normal morphology comparable to their controls (Figure 1Bd), providing evidence that melanin protects the epidermal structure of the skin equivalent against UV-induced damage.

### 3.2 UV-induced melanogenesis In irradiated pigmented skin equivalents

**Figure 2:**
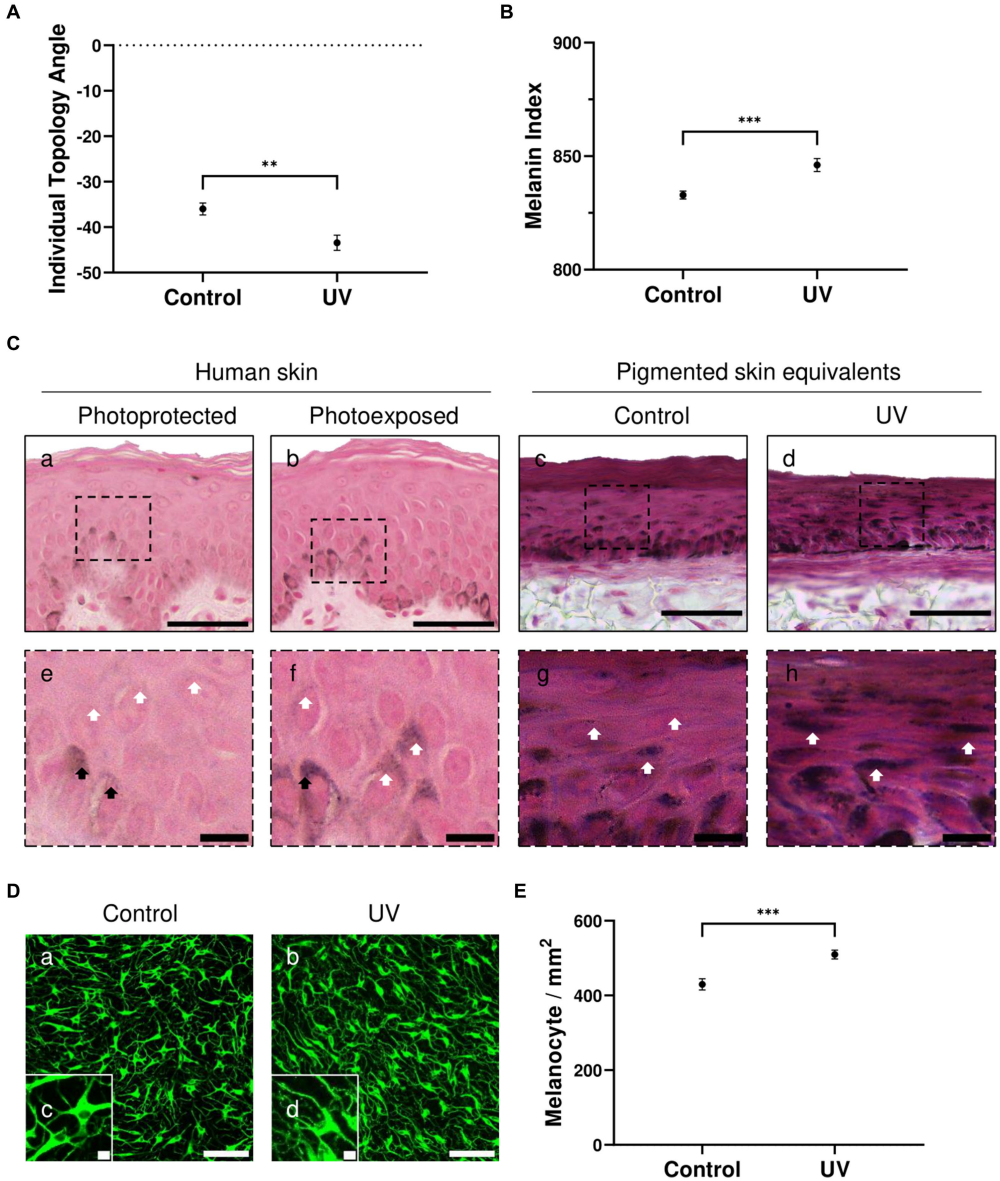
UV-induced hyperpigmentation in irradiated pigmented skin equivalents. Untreated and repeatedly irradiated pigmented skin equivalents were cultured for 21 days at ALI. UV-induced skin equivalents were irradiated with 3.3 J cm^−2^ (96.5% UVA and 3.5% UVB) for 5 consecutive days from day 15 to 19 at ALI and harvested 48 h later. Individual topology angle (ITA°) **(A)** and melanin index (MI) **(B)** show a significantly darker skin tone in irradiated pigmented skin equivalents compared to the control. Representative Fontana-Masson micrographs **(C)** of cross-sections of untreated **(Cc,g)** and repeatedly irradiated skin equivalents **(Cd,h)** and photoprotected **(Ca,e)** and photoexposed human skin **(Cb,f)** show melanocyte location in the *stratum basale* and melanin supranuclear cap formation in epidermal basal (black arrows) and suprabasal keratinocytes (white arrows). Increased melanin deposition is observed in repeatedly irradiated pigmented skin equivalents **(Cd)** compared to untreated pigmented skin equivalents **(Cc)**, consistent with the melanin supranuclear caps and higher melanin deposition present in photoexposed human skin **(Cb)** compared to photoprotected human skin **(Ca)**. Photoprotected and photoexposed skin samples from the buttock and arm sites, respectively, of a 22-year-old female, a Caucasian donor with Fitzpatrick SPT III, were provided by Procter and Gamble, Cincinnati, United States. Representative immunofluorescence analysis of melanocyte marker TYRP1 **(D)** demonstrates an even distribution of melanocytes within the basal layer of the pigmented skin equivalents. Immunofluorescence analysis of whole-mounted epidermises demonstrates a significant difference in melanocyte densities between control and irradiated conditions **(E)**. GraphPad Prism 9 software was used to generate the graphs displaying the mean ± SEM from ×3 independent experiments with ×3 skin equivalents per condition, with an unpaired, two-tailed *t*-test used to determine statistical significance, **p < 0.01, ***p < 0.001. Scale bars: 50 μm **(Ca–d)**, 10 μm **(Ce–h)**, 100 μm **(Da,b)**, 10 μm **(Dc,d)**.

ITA (Figure 2A) and MI (Figure 2B) demonstrate significant darker pigmentation when skin equivalents are repeatedly irradiated (average ITA = −43°, average MI = 846) compared to untreated pigmented skin equivalents (average ITA = −36°, average MI = 832).

Melanocytes residing in the epidermal basal layer and melanin supranuclear caps in epidermal basal and suprabasal keratinocytes are observed in Fontana Masson analysis of the pigmented skin equivalents (Figure 2Cc,d). Increased melanin deposition is observed in repeatedly irradiated pigmented skin equivalents (Figure 2Ch) compared to untreated pigmented skin equivalents (Figure 2Cg). This result is consistent with the increased number of melanin supranuclear caps and higher melanin deposition found in photoexposed human skin (Figure 2Cf) compared to photoprotected human skin (Figure 2Ce).

To determine whether melanocyte density increases in irradiated pigmented skin, epidermal whole-mount TYRP1 staining was performed in untreated and repeatedly irradiated pigmented skin equivalents and epidermises were imaged from the basal view to determine melanocyte densities. TYRP1 immunostaining shows a uniform melanocyte distribution and increased melanocyte dendricity when skin equivalents are irradiated (Figure 2D). The melanocyte density (Figure 2E) between conditions shows a significantly higher number in irradiated skin equivalents, which along with the increased dendricity could explain the higher melanin content.

These data indicate that the pigmented skin equivalents respond to UV exposure similarly to native human skin, and this stimulates melanogenesis and melanin transfer by increasing the number of melanocytes and their dendricity.

### 3.3 Epidermal apoptosis is increased when irradiated skin equivalents lack melanin

**Figure 3:**
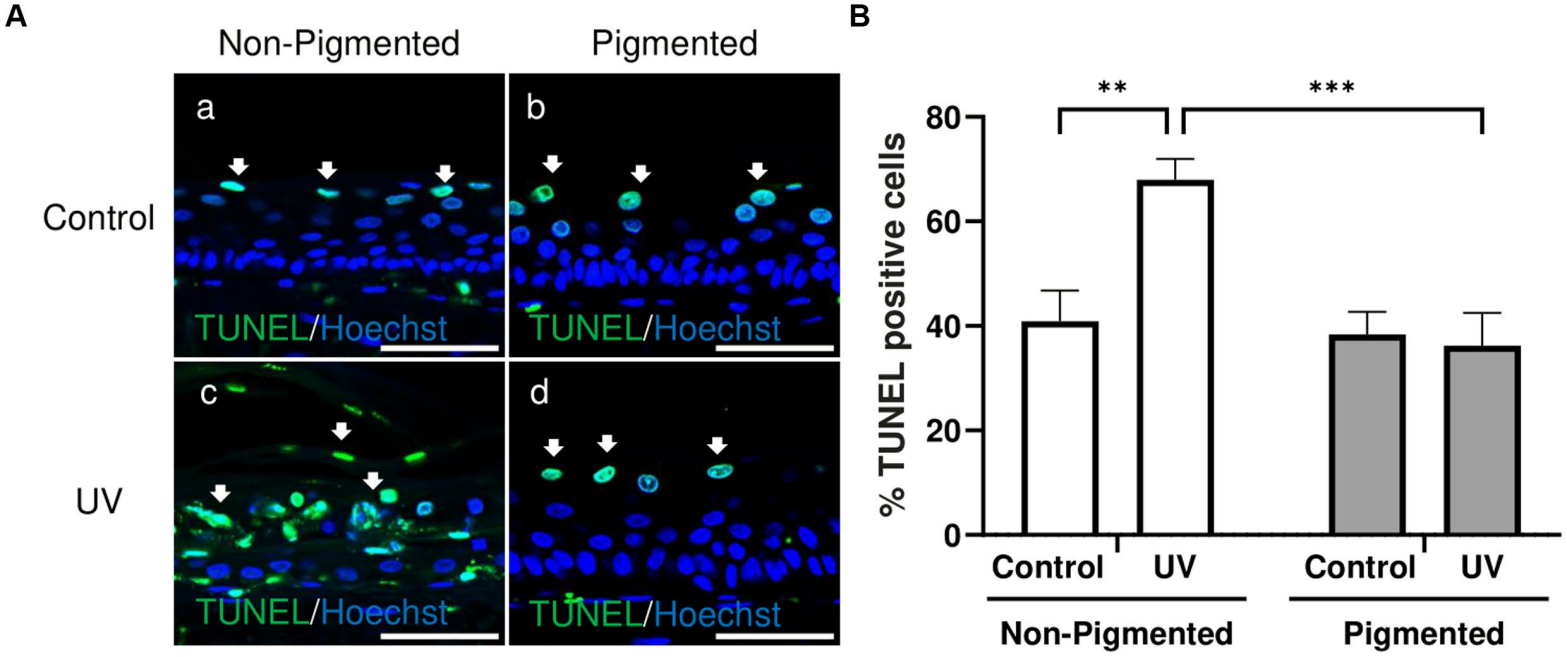
UV-induced epidermal apoptosis is only observed in irradiated non-pigmented skin equivalents. Representative immunofluorescence analysis of cross-sections of untreated and repeatedly irradiated skin equivalents cultured for 21 days at ALI. UV-induced skin equivalents were irradiated with 3.3 J cm^−2^ (96.5% UVA and 3.5% UVB) for 5 consecutive days from day 15 to 19 at ALI and harvested 48 h later. TUNEL immunolabeling in untreated and irradiated skin equivalents for identification of apoptotic cells **(A)** demonstrates apoptotic cells in suprabasal layers of the epidermis in controls **(Aa,b)** and irradiated pigmented skin equivalents **(Ad)**, whereas irradiated non-pigmented skin equivalents **(Ac)** display a higher number in different layers of the epidermis. % TUNEL positive cells of the epidermis from control and irradiated skin equivalents **(B)** show a higher number of apoptotic cells in irradiated non-pigmented skin equivalents compared to untreated non-pigmented skin equivalents and irradiated pigmented skin equivalents. GraphPad Prism 9 software was used to generate the graphs displaying the mean ± SEM from ×3 independent experiments with ×3 skin equivalents per condition. Ordinary two-way ANOVA with Tukey correction for multiple comparisons was used to determine statistical significance, **p < 0.01, ***p < 0.001. The nuclei are stained with Hoechst. Scale bars: 50  μm.

Changes in keratinocyte apoptosis were investigated by TUNEL assay. As demonstrated in Figure 3A, TUNEL-positive cells in control groups were located in the suprabasal layers of the epidermis, indicative of keratinocytes undergoing terminal differentiation and the start of cornification and keratinisation processes. Significant differences in the percentage of TUNEL-positive cells within the epidermis were observed with photoexposure in non-pigmented skin equivalents (Figure 3B). This is associated with the appearance of sunburn cells and parakeratosis observed previously in histology (Figure 1Bc). Sunburn cells represent structural change in irradiated skin, which are apoptotic vacuolated keratinocytes histologically found 24 h post-UV (64). However, no significant differences in the percentage of TUNEL-positive cells were found in irradiated pigmented skin equivalents compared to the control, which can be explained by the absence of morphological damage observed in histology (Figure 1Bf). These findings are consistent with the melanin role in photoprotection.

### 3.4 UV-induced epidermal DNA damage shows a different intensity and distribution in non-pigmented and pigmented skin equivalents

**Figure 4:**
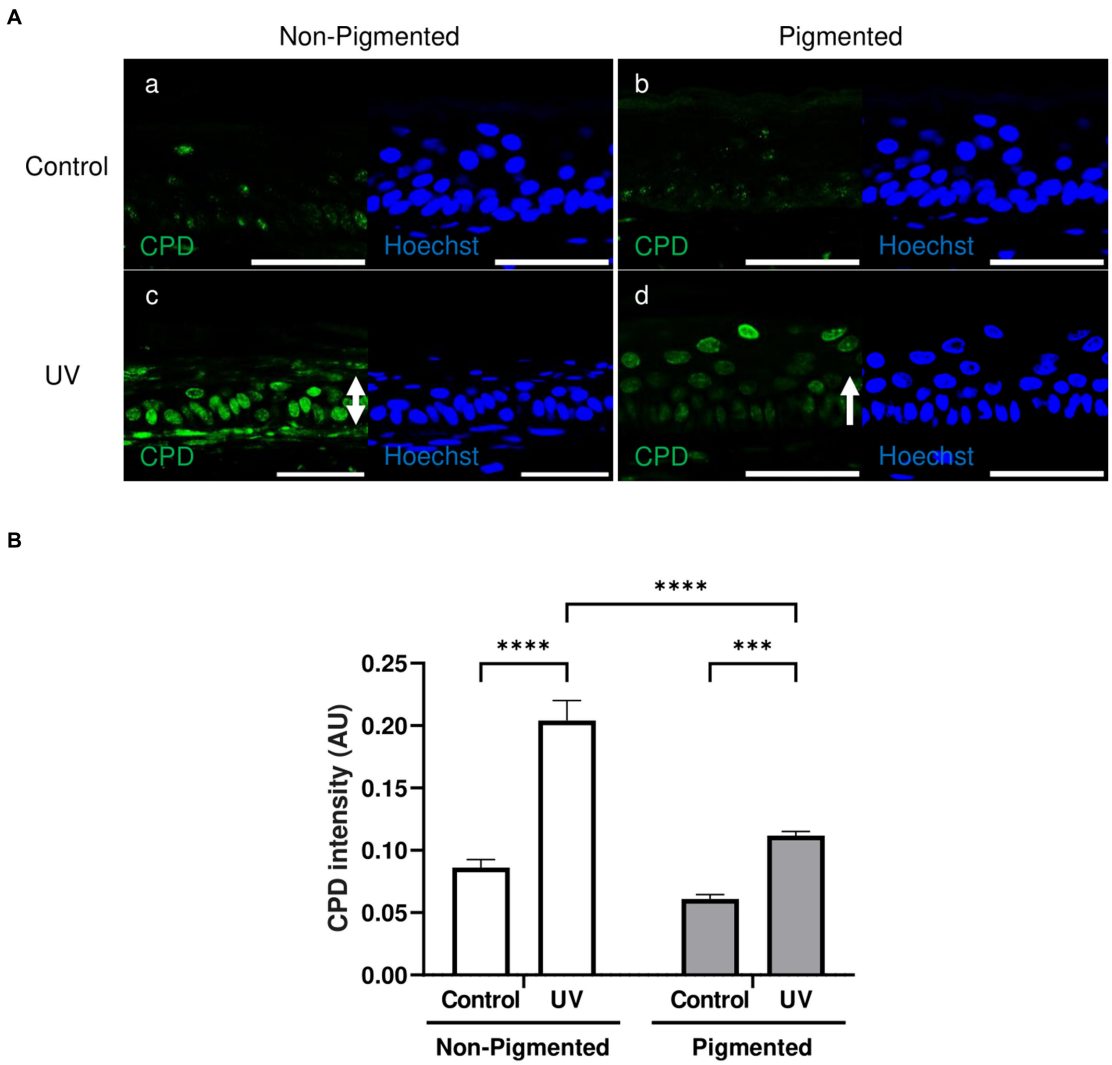
Epidermal cyclobutane pyrimidine dimer lesions in irradiated pigmented skin equivalents are significantly lower than in non-pigmented skin equivalents. Representative immunofluorescence analysis of cross-sections of untreated and repeatedly irradiated skin equivalents cultured for 21 days at ALI. UV-induced skin equivalents were irradiated with 3.3 J cm^−2^ (96.5% UVA and 3.5% UVB) for 5 consecutive days from day 15 to 19 at ALI and harvested 48 h later. Cyclobutane pyrimidine dimer (CPD) immunolabeling in untreated and irradiated skin equivalents **(A)** demonstrates a low level of DNA lesions in non-irradiated skin equivalents **(Aa,b)**, whereas irradiated skin equivalents display a higher number of positive cells **(Ac,d)** with a different intensity and distribution (white rows) between non-pigmented **(Ac)** and pigmented skin equivalents **(Ad)**. DNA damage in the epidermis of irradiated skin equivalents in the form of CPD lesions **(B)** is significantly different between irradiated skin equivalents and the untreated group. AU, arbitrary units. GraphPad Prism 9 software was used to generate the graphs displaying the mean ± SEM from ×3 independent experiments with ×3 skin equivalents per condition. Ordinary two-way ANOVA with Tukey correction for multiple comparisons was used to determine statistical significance, ***p < 0.001, ****p < 0.0001. The nuclei are stained with Hoechst. Scale bars: 50 μm.

A low CPD lesion level was found in the *stratum basale* in unexposed skin equivalents (Figure 4Aa,b). Increased CPD lesions were found in irradiated skin equivalents located throughout different epidermal layers (Figure 4Ac,d). However, DNA lesions in irradiated non-pigmented skin equivalents were strongly stained in all epidermal layers (Figure 4Ac). In contrast, irradiated pigmented skin equivalents showed a very weak intensity in the *stratum basale* and *stratum spinosum* compared to the stronger staining of the *stratum granulosum* (Figure 4Ad). Increased CPD intensities were found within the epidermis of irradiated skin equivalents compared to their controls (Figure 4B), but non-pigmented skin equivalents showed a significantly higher CPD intensity after UV exposure compared to the intensity in irradiated pigmented skin equivalents. This suggests that although DNA lesions are still produced in pigmented cells after UV exposure, these are fewer and localised mainly in suprabasal layers, suggesting the role of melanin in DNA protection in the lower epidermis.

### 3.5 Epidermal proliferation is not affected by repeated UV exposure in photoexposed pigmented skin equivalents

**Figure 5:**
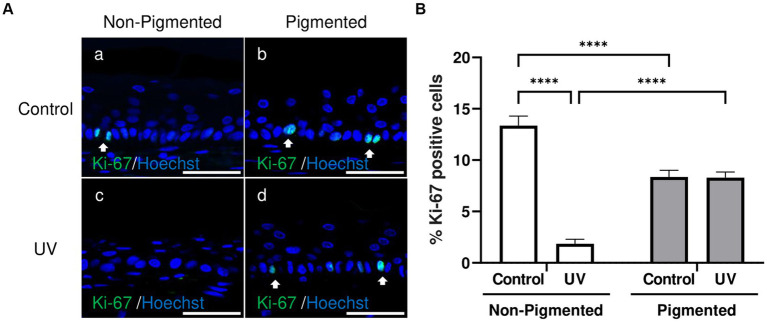
Epidermal proliferation is maintained in irradiated pigmented skin equivalents. Representative immunofluorescence analysis of cross-sections of untreated and repeatedly irradiated skin equivalents cultured for 21 days at ALI. UV-induced skin equivalents were irradiated with 3.3 J cm^−2^ (96.5% UVA and 3.5% UVB) for 5 consecutive days from day 15 to 19 at ALI and harvested 48 h later. Representative immunofluorescence micrographs of proliferation marker Ki-67 **(A)**. % Ki-67 positive cells of the epidermis from control and irradiated skin equivalents **(B)** show a lower proliferation in irradiated non-pigmented skin equivalents compared to non-irradiated skin equivalents and lower levels of proliferation when melanocytes are incorporated into the epidermis. GraphPad Prism 9 software was used to generate the graphs displaying the mean ± SEM from ×3 independent experiments with ×3 skin equivalents per condition. Ordinary two-way ANOVA with Tukey correction for multiple comparisons was used to determine statistical significance, ****p < 0.0001. The nuclei are stained with Hoechst. Scale bars: 50 μm.

Ki-67, a classical marker of cellular proliferation, was measured to investigate UV-induced effects in keratinocyte proliferation. As demonstrated in Figure 5A, Ki-67 positive cells were located in the epidermal basal layer in untreated and exposed skin equivalents. Significant differences in the percentage of Ki-67 positive cells within the epidermis were observed with photoexposure in non-pigmented skin equivalents (Figure 5B). In contrast, no significant differences in the percentage of Ki-67 positive cells were found after repeated UV exposure to pigmented skin equivalents. Interestingly, a significant difference in the percentage of Ki-67 positive cells was observed between unexposed non-pigmented and pigmented skin equivalents (Figure 5B).

### 3.6 Irradiated skin equivalents show an inflammatory response during repeated UV exposure

**Figure 6:**
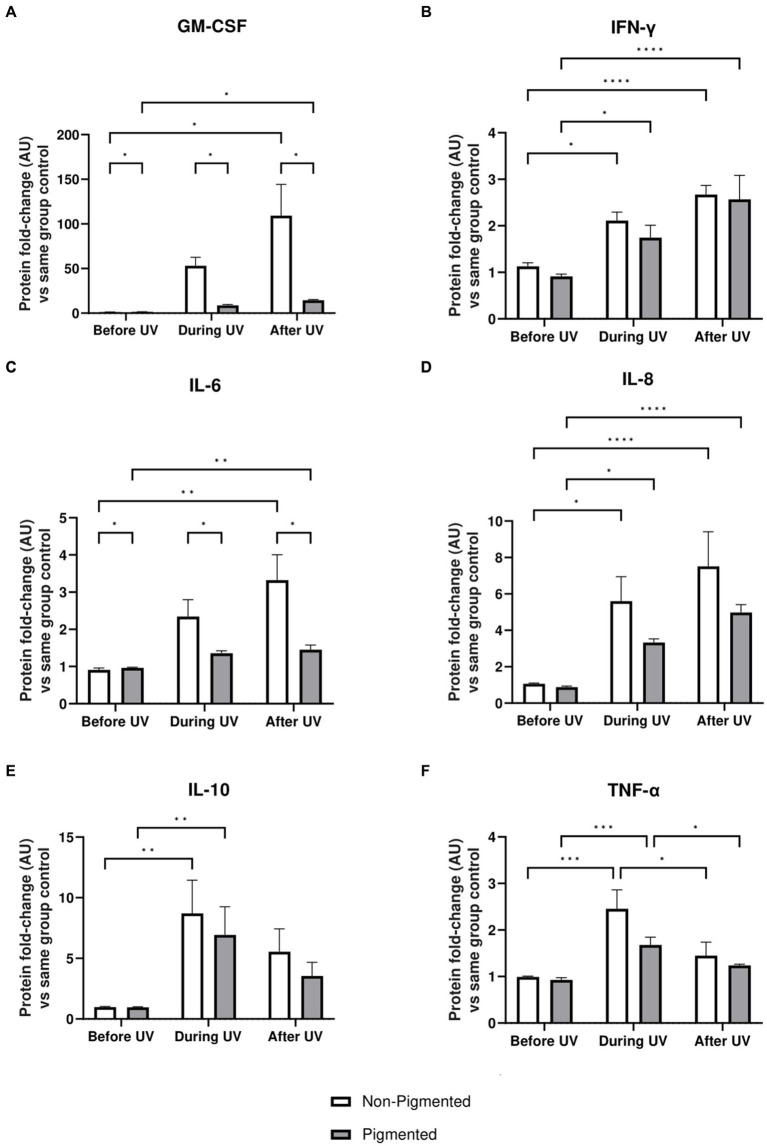
Inflammatory response after UV exposure in irradiated pigmented and non-pigmented skin equivalents. Untreated and repeatedly irradiated skin equivalents were cultured for 21 days at ALI. UV-induced skin equivalents were irradiated with 3.3 J cm^−2^ (96.5% UVA and 3.5% UVB) for 5 consecutive days from day 15 to 19 at ALI and harvested 48 h later. Culture medium was taken at days 15, 18 and 21 of ALI, corresponding to days 1 (before the 1st UV dose) and 4 (during UV/before the 4th UV dose) of irradiation and 2 days after the last UV exposure. Culture medium from untreated non-pigmented and pigmented skin equivalents at days 15, 18 and 21 of ALI was considered as control of each independent experiment. The average of control samples was adjusted to 1. Values from the supernatant of irradiated skin equivalents were divided by the average concentration of the control at the same time. GM-CSF **(A)**, IFN-γ **(B)**, IL-6 **(C)**, IL-8 **(D)**, IL-10 **(E)** and TNF-α **(F)** secretion was increased in non-pigmented and pigmented skin equivalents during UV exposure. Only IL-10 **(E)** and TNF-α **(F)** secretion was recovered 2 days after the last UV exposure, while the rest were increased. GM-CSF **(A)** and IL-6 **(C)** secretion was significantly upregulated in irradiated non-pigmented skin equivalents compared to pigmented skin equivalents. GraphPad Prism 9 software was used to generate the graphs displaying the mean ± SEM from ×2 independent experiments with ×3 technical repeats per condition per time point. Ordinary two-way ANOVA with Tukey correction for multiple comparisons was used to determine statistical significance, *p ≤ 0.05, **p ≤ 0.01, ***p ≤ 0.001, ****p ≤ 0.0001. AU, arbitrary units.

Inflammation profiles of irradiated skin equivalents were analysed. GM-CSF, IFN-γ, IL-6, IL-8, IL-10, and TNF-α showed a significantly higher secretion during and/or after UV exposure, demonstrating an inflammation response (Figure 6). In addition, the average secretion of these markers was higher in irradiated non-pigmented skin equivalents, with a significant increase in GM-CSF (Figure 6A) and IL-6 secretion (Figure 6C). IL-10 (Figure 6E) and TNF-α (Figure 6F) showed lower secretion during the recovery time, with only TNF-α significantly decreasing. These results show the induction of different signalling pathways of inflammation in irradiated skin equivalents, which recapitulate UV-induced inflammation of native human skin.

### 3.7 Irradiated skin equivalents show ECM remodelling response, one of the major contributions of UVA radiation

**Figure 7:**
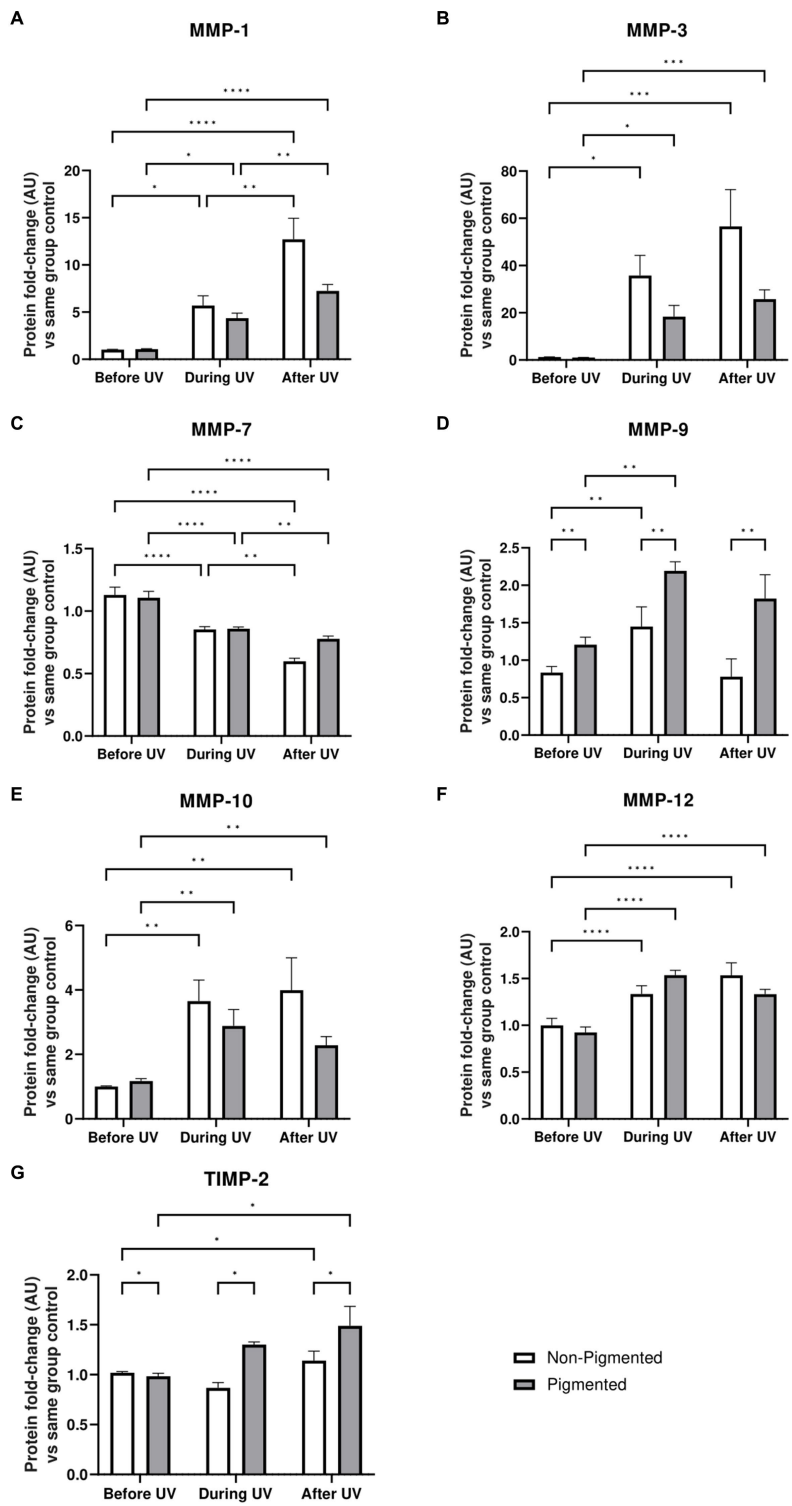
ECM-remodelling response after UV exposure in irradiated pigmented and non-pigmented skin equivalents. Untreated and repeatedly irradiated skin equivalents were cultured for 21 days at ALI. UV-induced skin equivalents were irradiated with 3.3 J cm^−2^ (96.5% UVA and 3.5% UVB) for 5 consecutive days from day 15 to 19 at ALI and harvested 48 h later. Culture medium was taken at days 15, 18 and 21 of ALI, corresponding to days 1 (before the 1st UV dose) and 4 (during UV/before the 4th UV dose) of irradiation and 2 days after the last UV exposure. Culture medium from untreated non-pigmented and pigmented skin equivalents at days 15, 18 and 21 of ALI was considered as control of each independent experiment. The average of control samples was adjusted to 1. Values from the supernatant of irradiated skin equivalents were divided by the average concentration of the control at the same time. MMP-1 **(A)**, MMP-3 **(B)**, MMP-9 **(D)**, MMP-10 **(E)**, MMP-12 **(F)** and TIMP-2 **(G)** secretion was increased in non-pigmented and pigmented skin equivalents during UV exposure. Only MMP-7 **(C)** showed a decrease with UV exposure. MMP-9 **(D)** and TIMP-2 **(G)** secretion was significantly upregulated in irradiated pigmented skin equivalents compared to non-pigmented ones. GraphPad Prism 9 software was used to generate the graphs displaying the mean ± SEM from ×2 independent experiments with ×3 technical repeats per condition per time point. Ordinary two-way ANOVA with Tukey correction for multiple comparisons was used to determine statistical significance, *p ≤ 0.05, **p ≤ 0.01, ***p ≤ 0.001, ****p ≤ 0.0001. AU, arbitrary units.

Under the conditions we tested, such ECM changes were not detected by immunostaining of irradiated skin equivalents after ×5 daily UV exposures. However, significant differences in MMP and TIMP secretion in conditioned medium were identified during the irradiation of skin equivalents. Protein-level analysis was conducted on the conditioned medium of skin equivalents collected before, during, and after UV exposure. MMP-1, MMP-3, MMP-9, MMP-10 and MMP-12 levels in the culture medium are significantly increased during UV exposure (Figure 7). All the previous markers, except MMP-9 and TIMP-2, showed significantly higher secretion after UV exposure. MMP-9 and TIMP-2 levels significantly increased in pigmented skin equivalents during UV exposure compared to in non-pigmented skin equivalents. In contrast, MMP-7 secretion was significantly decreased during UV exposure and after recovery time in both non-pigmented and pigmented skin equivalents.

## 4 Discussion

UV solar radiation is well studied due to its high energy and biological action on the skin. Although skin equivalents have previously been used to characterise the effect of UV radiation on skin, the lack of an integral dermal layer capable of endogenous ECM production, crosstalk between fibroblasts and melanocytes, and the use of non-physiological relevant doses of UV radiation do not contribute to recapitulating the characteristics of irradiated human skin *in vitro*. In this study, we utilised a human pigmented full-thickness skin equivalent that recapitulates the physiological microanatomy of native human skin with supranuclear melanin caps within the keratinocytes (56). This bioengineered construct represents an improved platform for studying biological end-points subsequent to UV exposure. We compared these to the response induced in non-pigmented full-thickness skin equivalents to investigate the role of melanocytes in the UV response. Using a controlled physical dosage of UV provided an advantage for comparing of responses to the same UV dose in different skin equivalents. In addition, a UVA/UVB ratio of 27 represents a real daylight value (65) compared to other UV-induced skin equivalents where a lower UVA/UVB ratio was used. Furthermore, skin equivalents were irradiated for 5 consecutive days, which better resembles repeated exposure experienced *in vivo* compared with a single exposure, as human skin may be photoexposed daily.

UV-induced skin equivalents exhibited typical histological alterations identified in the epidermis of human skin exposed to single or repeated UV exposures. Sunburn cell formation is a feature of erythema response well characterised in irradiated human epidermis (4–10, 66–69), along with epidermal thickness changes (6, 9, 10, 21, 70, 71). Epidermal thinning has been observed *in vitro* after single or repeated UV exposure to non-pigmented skin equivalents from 24 to 72 h post-UV (37, 38, 72). In the present study, the equivalent UV exposure was also delivered to pigmented full-thickness skin equivalents, which was insufficient to induce the same level of UV-induced epidermal damage in non-pigmented skin models. A similar result was shown by comparison of non-pigmented and pigmented epidermal equivalents after a single UVB exposure of 0.150 J cm^−2^, where melanin demonstrated protection against sunburn induction (73). Furthermore, previous data using non-pigmented epidermal equivalents demonstrated that single irradiation of 0.250 J cm^−2^ induced epidermal damage (Supplementary Figure S1). When a higher dose (3.3 J cm^−2^) was used for single irradiation of non-pigmented full-thickness skin equivalents, only sunburn cells were identified, and greater epidermal damage was shown when the exposure was repeated for 5 days (Supplementary Figure S2). This suggests that the dermal compartment, along with the pigmentation, plays a role to withstand higher or repetitive UV doses.

We have presented evidence that changes in darkening pigmentation, melanin synthesis, melanin distribution, and melanocyte density observed in photoexposed native human skin were produced in the irradiated pigmented skin equivalents presented herein. The induction of facultative pigmentation in human skin has been characterised after acute (4, 11–13, 16, 19, 74–77) and chronic irradiation (9, 10, 13, 19–21, 25, 69, 71) at different time points by colourimetric measurements, melanin quantification, evaluation of melanin distribution in Fontana Masson, melanocyte density and expression of melanocyte-specific related proteins. Changes in ITA and melanin index reflected a darkening pigmentation in our skin equivalents after repeated UV exposure, which was also observed macroscopically as the skin model darkened in tone. Although other irradiated full-thickness skin equivalents have shown tanning induction by colourimeters measurements (53) and higher melanin content from image analysis (12, 78), only Duval et al. (32, 79) have reported macroscopic images of UV-induced pigmentation in epidermal equivalents. According to the different phases of darkening responses characterised *in vivo* after UV exposure (80), the response observed in our skin equivalents corresponds to delayed tanning, which involves melanin neo-synthesis and does not occur immediately after UV exposure but 3 to 5 days later.

Irradiated pigmented skin equivalents displayed UV-induced melanocyte proliferation as observed in photoexposed human skin. Our results showed a significantly higher melanocyte density 48 h after repeated UV exposure and an increased dendritic morphology. This is consistent with studies of chronic UV exposure on human skin, which have demonstrated induction of a significantly higher number of melanocytes during the first days after different UV exposures (13, 81–84). Moreover, this is also reflected in studies of chronologically photoexposed human skin sites and photoprotected areas, where significantly higher melanocyte density has been reported in photoexposed sites (85–87). A higher melanocyte number after UV exposure has not yet been reported in pigmented skin equivalents.

The proportion of TUNEL-positive cells were found to be significantly higher 48 h after UV exposure only in non-pigmented skin equivalents compared to the untreated group. The UVB induction of apoptosis, which is shown to be related to the sunburn cell formation, represents a mechanism of efficient removal of UV-damaged cells. Significantly higher numbers of TUNEL-positive cells have been identified *in vivo* following chronic UV exposure compared to untreated sites (13), although the numbers reported were noted to be low in order to be physiologically relevant. In addition, Del Bino et al. (4) described that darker human skin types *ex vivo* showed rare or undetected caspase-3 positive cells (a marker of cells undergoing early stages of apoptosis) 24 h after a single UV exposure, when compared to lighter skin types, even when the UV dose used in darker skin types was higher. Yamaguchi et al. (12) had previously shown that increased levels of melanin correlated directly with the number of TUNEL-positive cells in single irradiated pigmented epidermal equivalents (MelanoDerm) of different pigmentations 48 h following single UV exposure (0.009 J cm^−2^ UVA and 0.0016 J cm^−2^ UVB, and 0.0018 J cm^−2^ UVA and 0.032 J cm^−2^ UVB). Although the single UVB dose is 3.6 to 7.2 times lower than the daily dose used in our skin equivalents (0.115 J cm^−2^ UVB), our findings showed more TUNEL-positive cells were found in non-pigmented skin equivalents compared to pigmented skin equivalents after repeated UV exposure. The significant changes observed in irradiated darkly pigmented epidermal equivalents with a low dose, compared to the dose used in our study, emphasise the need for a more complex skin equivalent that includes the interactions of the dermis and epidermis. In turn, this further justifies the need to use more physiologically relevant full-thickness skin equivalents that produce data that align more consistently with changes observed in real human skin (88).

DNA damage plays a crucial role in photocarcinogenesis, one of the most well-documented consequences of UV exposure. UV radiation of human skin can produce two major types of DNA lesions: 6,4-photoproducts (6,4 PP) and cyclobutane pyrimidine dimers (CPD) (12). These lesions have been identified in irradiated human skin and can be detected in the first minutes after a single UV exposure (11). It has been shown that CPD is the primary DNA lesion, but 6,4-photoproducts are repaired faster, as shown *in vivo* (11, 12) and *in vitro* (73). Epidermal CPD lesions were higher in our irradiated skin equivalents, with a significant difference between non-pigmented and pigmented skin equivalents. Irradiated pigmented skin equivalents showed a stronger distribution of the CPD lesion towards the upper epidermal layers with fewer lesions in the low epidermis. This suggests pigmentation does not avoid DNA damage, but the level of damage can be related to the melanin content. It has been shown that epidermal cells with supranuclear melanin caps have significantly fewer DNA photoproducts than epidermal cells without supranuclear melanin caps *in vivo* (89), which explains that although CPD lesions are still observed in cells with melanin, DNA damage within the cell appears less. UV-induced CPD formation in human skin has also been reported when using suberythemal doses, which did not induce sunburn cell formation and showed that epidermal CPD formation was dose-dependent, that CPD lesions accumulated in repetitive exposure, and that they are associated with the physical dose regardless of the skin type; although the distribution is dependent of the skin pigmentation (4, 7, 69). Similar trends have been seen *in vitro* after single UV exposure (12, 73, 78). Cario-André et al. (73) showed that the percentage of epidermal CPD-positive cells 24 h following a single UVB exposure was not different between non-pigmented and pigmented epidermal equivalents. Yamaguchi et al. (12) demonstrated that epidermal CPD lesions correlate negatively with melanin content in pigmented epidermal equivalents, while differences in CPD distribution and CPD fluorescence intensity in UV-induced non-pigmented and pigmented full-thickness skin equivalents after acute UV exposure were described by Goyer et al. (78). Our data support that similar trends are observed in repeatedly irradiated full-thickness skin equivalents.

As changes in epidermal proliferation are characteristic of irradiated human skin, we examined this in our skin equivalents. The reduced epidermal proliferation observed in our irradiated non-pigmented skin equivalents is consistent with other studies where epidermal proliferation has been reduced the first days after acute exposure to UVA (90) and UVB radiation (29) in non-pigmented epidermal equivalents or UV exposure to full-thickness skin equivalents (72) by decreasing the percentage of Ki-67 positive cells or downregulation of gene expression. However, our study supports that including melanocytes can prevent this lower proliferation rate when using the same UV doses in non-pigmented full-thickness skin equivalents. *In vivo*, epidermal proliferation is dose-dependent in chronic UV exposure (6). We hypothesise that the lower epidermal proliferation is only observed in our non-pigmented skin equivalents after repeated exposure due to the greater epidermal histological changes, apoptosis, and DNA damage seen in irradiated non-pigmented skin equivalents as a possible mechanism to provide time for DNA repair. Interestingly, we observed a significant decreased epidermal proliferation in unexposed skin equivalents when melanocytes were incorporated compared to the non-pigmented ones (ca. 13.4%). The lower values described in non-exposed and irradiated pigmented skin equivalents (ca. 8.4 and 8.3%) are closer to the percentage of Ki-67 positive cells reported in human skin (4.1–5.9%) (58), which could indicate a melanocyte role in the epidermal proliferation rate.

We also analysed the secretion of inflammatory proteins in our UV-induced skin equivalents to investigate the response in repeated irradiation. Immunosuppression (reduced number of immune cells in the epidermis and morphological alterations), immune cell infiltration and release of a cascade of inflammatory mediators are well-documented characteristics of the inflammatory response induced after acute and chronic UV exposure *in vivo* (9, 20, 91). In addition, low chronic level inflammation from environmental UV exposure strongly influences on the ageing process, which has been shown by the higher cytokine levels in chronically photoaged skin compared to chronically aged skin (92). Our data supports evidence of an inflammation response during and after UV exposure in both systems. Some studies have shown upregulation of inflammatory molecules after acute UVB exposure to non-pigmented epidermal skin equivalents (29, 31), and UVA exposure to non-pigmented full-thickness skin equivalents (15, 27, 76, 93), indicating both UVA and UVB are likely to contribute to an inflammatory response. In addition, there is evidence of upregulation of inflammatory genes by keratinocytes and fibroblasts in non-pigmented full-thickness skin equivalents after acute exposure, demonstrating that both cell types contribute to the UV-induced immune response (72). Although we have also shown a higher cytokine secretion during UV exposure, our data shows different responses 2 days after the last irradiation with a decrease in anti-inflammatory cytokine IL-10 (*p* > 0.05) and pro-inflammatory cytokine TNF-α (*p ≤ 0.05). This study also provides evidence of the melanocyte role in the inflammatory response, as the mean cytokine levels are higher in irradiated non-pigmented skin equivalents than those described by pigmented skin equivalents, with significantly elevated GM-CSF and IL-6 secretion. It is interesting to highlight that although our skin equivalents do not include immune cells, similar results were obtained in comparison with an irradiated immunocompetent skin equivalent, where higher IL-6, IL-8 and lower IL-10 levels were detected in culture medium 48 h after acute irradiation (94). Future directions to investigate the immune response to UV exposure include introducing immune cells into our skin equivalents to study their contribution to the inflammatory response to exogenous stressors.

Cytokines that are significantly regulated in our UV-induced skin equivalents during and after repeated UV exposure are associated with different mechanisms such as pigmentation, apoptosis, DNA repair and senescence. GM-CSF is known to act as a pro-pigmenting agent after UV exposure by regulating MITF and melanogenesis enzymes transcription through different signal transduction pathways, while IL-6, along with other factors such as TNF-α, suppresses skin pigmentation (95). GM-CSF, TNF-α, IL-6 and IL-8 are pro-inflammatory proteins which are involved in response to ROS production and DNA damage produced by UV exposure, while IL-10 is a potent anti-inflammatory cytokine, which plays a major role in regulating immune responses and maintaining skin homeostasis (94, 96).

Alongside inflammation, one well-known effect of chronic UV exposure in the dermis is ECM remodelling through MMP regulation. Acute and chronic UV exposure to human skin have demonstrated changes in the dermis, which could be linked to initial stages of photoageing (6, 20, 21, 70, 97). Dermal morphological changes and MMP upregulation have been demonstrated in photoaged human skin sites compared to intrinsically aged skin sites (98–100) and after acute and chronic UV exposure to human skin *in vivo*, mainly attributed to the UVA effect (15, 16, 19, 20, 101).

Upregulation of matrix metalloproteinases (MMP) and damage to the ECM, such as collagen degradation and accumulation of elastin fragments are hallmarks of long-term UV exposure (20). Significant changes in ECM deposition, such as collagen expression, could not be identified by immunostaining in our irradiated skin equivalents, which could be due to the low number of exposures over a relatively short period. However, by analysis of the MMP and TIMP secretion from the conditioned medium, MMPs were significant upregulated during and after UV exposure (Figure 7). Upregulation of MMP-1, MMP-3 and MMP-9 have been described *in vitro* in single UV-induced non-pigmented full-thickness skin equivalents (15, 27, 34, 35, 72, 76, 90, 93), and MMP-1 upregulation after repeated UV exposure to non-pigmented full-thickness skin equivalents (37, 38). Our study demonstrates higher secretion of MMP-3, MMP-9, MMP-10 and MMP-12 during UV exposure and higher levels of TIMP-2 after UV exposure, regardless of including melanocytes in the model. However, when comparing both systems, irradiated pigmented skin equivalents showed a higher MMP-9 and TIMP-2 secretion than non-pigmented skin equivalents. The influence of epidermal melanin content in ECM remodelling proteins during UV exposure to skin *in vitro* has not been described yet in the literature; however, this approach represents a useful platform to investigate the impact of skin colour on the dermal changes induced after UV exposure.

We have provided evidence of UV-induced changes in the dermal compartment of our photoexposed skin equivalents. MMP-1 is a common photoaging biomarker and major enzyme involved in the collagenolytic process, degrading interstitial collagen type I and II, MMP-3 degrades collagen I, MMP-7 and MMP-12 degrade elastin, MMP-9 promotes the degradation of collagen type IV, a major component of the basement membrane, and MMP-10 activates pro-MMPs (102).

## 5 Conclusion and future directions

We have developed an *in vitro* platform that recapitulates different aspects of UV exposure in human skin. Our robust and reproducible skin equivalents uniquely include human fibroblast-derived dermal matrix, human melanocytes, and supranuclear melanin caps, which evidently respond to UV light exposure in a manner compatible with native human skin. The comparative study and comprehensive characterisation between non-pigmented and pigmented skin equivalents after repeated UV exposure provide insights into the melanocyte role in the protection against UV-induced damage. We have simulated repeated UV exposure and shown that including melanocytes in the skin equivalent prevents epidermal thinning, parakeratosis, increased epidermal apoptosis, reduced epidermal proliferation, and a significant increase in epidermal CDP lesions. In addition, this study has reported upregulation of ECM remodelling and inflammatory proteins, demonstrating a response from keratinocytes and fibroblasts during and after repeated irradiation exposure, and noted differences between non-pigmented and pigmented skin equivalents. Furthermore, UV-induced melanogenesis, increased melanocyte density and dendricity were described in our pigmented skin equivalents as observed in native skin.

This characterisation *in vitro* provides cellular and molecular insight into *in vivo* events during UV exposure to human skin and offers an in-depth analysis of different alterations induced by UV exposure. This platform can be applied to a range of industrial and academic pursuits. The pigmented skin equivalent research tool could be used to gain fundamental insights into the molecular processes, specific markers and signalling pathways of the various effects of UV radiation on human skin. Skin equivalents can be tailored according to the research needs to determine the individual contribution of different cell types, such as the melanocyte contribution to the UV response described in this study. This platform also provides a pre-clinical tool for testing cosmetic actives designed to prevent or treat the deleterious effects of repeated UV exposure, such as sunscreen and after-sun products.

Future directions of this work include further characterisation of the UV-induced skin equivalents, such as gene expression and protein quantification of relevant dermal biomarkers, and the study of ageing hallmarks, such as cellular senescence and oxidative stress-specific markers present in the epidermis and dermis. The development of this platform might lead to the investigation of DNA damage and repair in different skin pigmentations, fluence dependance and the individual contributions of UVA and UVB irradiation to the changes described in this study. Finally, the addition of immune cells in the epidermal and dermal compartments will further enhance the *in vivo* human skin structure more accurately and better understand the responses induced by solar UV exposure and their role in inflammation homeostasis in human skin.

## Data availability statement

The raw data supporting the conclusions of this article will be made available by the authors, without undue reservation.

## Ethics statement

The studies involving humans were approved by Department of Biosciences, Ethics Committee, Durham University. The studies were conducted in accordance with the local legislation and institutional requirements. The participants provided their written informed consent to participate in this study.

## Author contributions

PD: Conceptualization, Data curation, Formal analysis, Investigation, Methodology, Project administration, Visualization, Writing – original draft. LC: Investigation, Methodology, Validation, Writing – review & editing. KG: Investigation, Methodology, Validation, Writing – review & editing. SP: Conceptualization, Funding acquisition, Project administration, Resources, Supervision, Writing – review & editing.
