# Supplementary figures and images for: Comparison of photodamage in non-pigmented and pigmented human skin equivalents exposed to repeated ultraviolet radiation to investigate the role of melanocytes in skin photoprotection
Source: Front Med (Lausanne). 2024 Apr 18;11:1355799. doi: 10.3389/fmed.2024.1355799 (PMC11063240; doi:10.3389/fmed.2024.1355799)

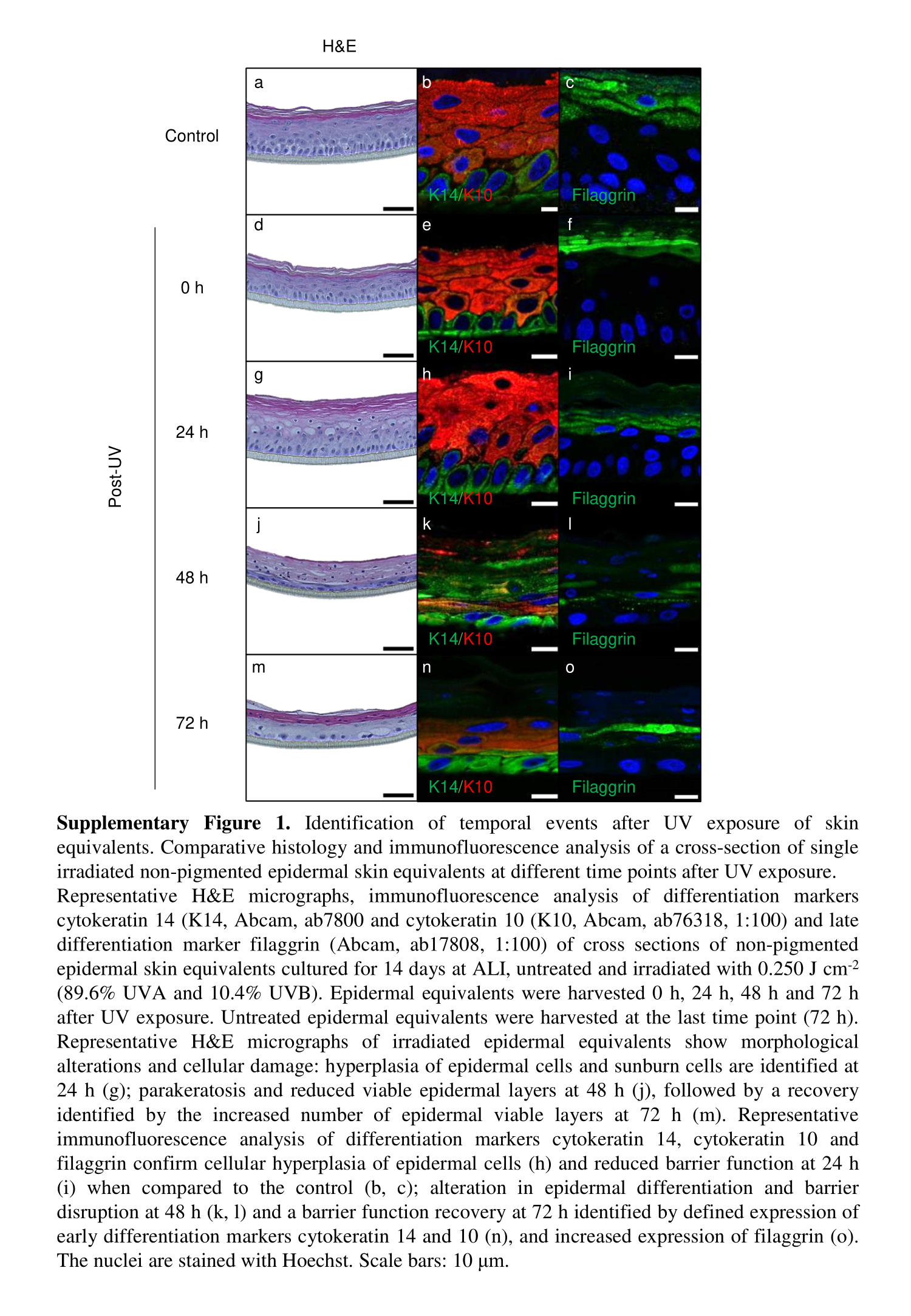

Supplement: Supplementary file 1 [file Image_1.TIFF]

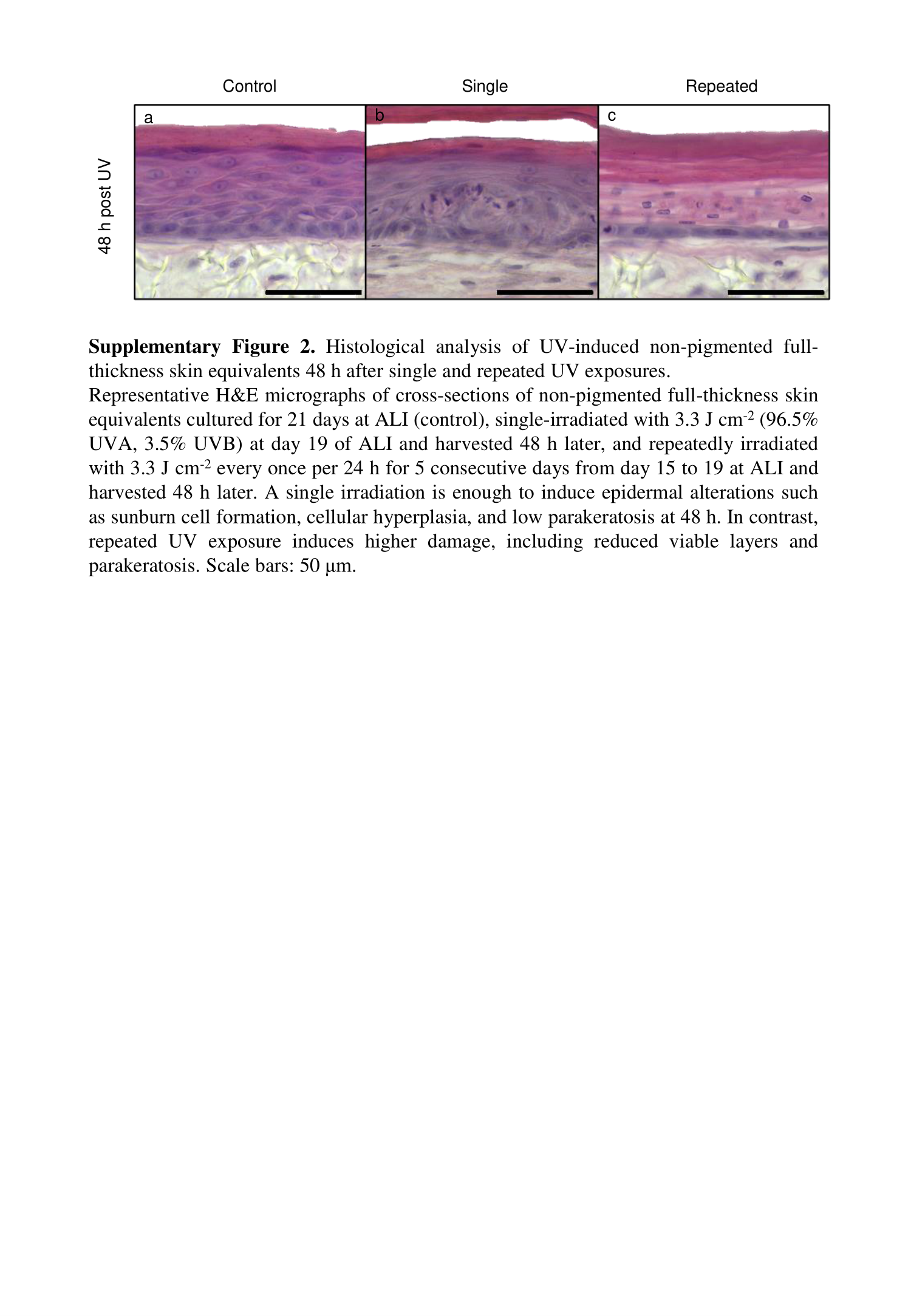

Supplement: Supplementary file 2 [file Image_2.TIFF]
